# Office workers’ experiences of attempts to reduce sitting-time: an exploratory, mixed-methods uncontrolled intervention pilot study

**DOI:** 10.1186/s12889-019-7196-0

**Published:** 2019-06-25

**Authors:** Stephen Dewitt, Jennifer Hall, Lee Smith, John P. Buckley, Stuart J. H. Biddle, Louise Mansfield, Benjamin Gardner

**Affiliations:** 1 Department of Psychology, King’s College London, London, UK; 2 Department of Experimental Psychology, University College London, London, UK; 3 Bradford Institute for Health Research, Bradford Teaching Hospitals NHS Foundation Trust, Bradford, UK; 4 The Cambridge Centre for Sport and Exercise Sciences, Anglia Ruskin University, Cambridge, UK; 5 The Centre for Active Living, University Centre Shrewsbury, Shrewsbury, UK; 6 University of Southern Queensland, Springfield, Australia; 7 Department of Life Sciences, College of Health and Life Sciences, Brunel University, Uxbridge, UK

**Keywords:** Sedentary behaviour, Workplace, Qualitative, Occupational health

## Abstract

**Background:**

Office workers typically sit for most of the workday, which has been linked to physical and mental ill-health and premature death. This mixed-methods study sought to identify barriers and facilitators to reducing sitting and increasing standing among office workers who received an intervention prototype (the ‘ReSiT [Reducing Sitting Time] Study’). The intervention comprised a sit-stand workstation and tailored advice to enhance motivation, capability and opportunity to displace sitting with standing.

**Methods:**

Twenty-nine UK university office workers (aged ≥18y, working ≥3 days per week, most time spent at a seated desk) participated in a 13-week uncontrolled study. They were initially monitored for one-week. In a subsequent face-to-face consultation, participants received sitting time feedback from a prior one-week monitoring period, and selected from a set of tailored sitting-reduction techniques. Quantitative data comprising sitting, standing and stepping time, which were objectively monitored for 7 consecutive days across three post-intervention timepoints, were descriptively analysed. Qualitative data, from semi-structured interviews conducted at 1, 6 and 12-weeks post-intervention, were thematically analysed.

**Results:**

Compared to baseline, mean sitting time decreased at weeks 1, 6 and 12 by 49.7mins, 118.2mins, and 109.7mins respectively. Despite prior concerns about colleagues’ reactions to standing, many reported encouragement from others, and standing could be equally conducive to social interaction or creating private, personal space. Some perceived less cognitively-demanding tasks to be more conducive to standing, though some found standing offered a valued break from challenging tasks. Participants prioritised workload over sitting reduction and were more likely to stand after rather than during work task completion. Temporary context changes, such as holidays, threatened to derail newfound routines.

**Conclusions:**

Our findings emphasise the importance of understanding workers’ mental representations of their work, and the social functions of sitting and standing in the workplace. Workplace intervention developers should incorporate a pre-intervention sitting time monitoring period, encourage workers to identify personally meaningful tasks and cues for standing, and build organisational support for sitting-reduction. We will use these insights to refine our intervention for self-administered delivery.

**Trial registration:**

ISRCTN29395780 (registered 21 November 2016).

**Electronic supplementary material:**

The online version of this article (10.1186/s12889-019-7196-0) contains supplementary material, which is available to authorized users.

## Background

Prolonged sitting is associated with poor mental and physical health and premature death [1–4]. Office workers typically sit for two-thirds of their waking day, so are at particular risk [5, 6]. Offsetting this risk requires displacing sitting with standing or light activity. Expert guidance recommends that workers regularly break up sitting and accumulate 2-4 h standing per 8 h workday [7]. Sitting-reduction interventions are needed to achieve these targets.

Successful implementation of such interventions depends upon acknowledging the complex organisational, social and cultural factors that shape the modern workplace [8]. Some workplace sitting-reduction approaches show efficacy yet lack acceptability, because they fail to address the needs and priorities of organisations or their employees. For example, automated prompts to stand, delivered at fixed intervals (e.g. hourly), can reduce sitting time [9], but some workers report dissatisfaction because prompts disrupt their workflow [10]. Similarly, workers who volunteered to stand felt unable to fully engage in otherwise-seated meetings [11]. Interventions that adversely impact productivity are unlikely to be acceptable to employees or managers [12].

Height-adjustable sit-stand workstations (SSWs) allow alternation between sitting and standing while working, so are viewed favourably by workers [13, 14]. SSWs take two forms: sit-stand desks allow adjustment of the entire desk-top surface and are costly, whereas desk-mounted sit-stand units adjust only monitor and keyboard height and are relatively inexpensive. Both can reduce sitting time: although trials have typically been of low quality, SSWs reduce sitting by around 100mins per 8 h workday [9], with effects persisting over time [9, 15–19]. Yet, 100mins reduction in sitting may fall short of achieving 2-4 h standing time. Supplementing SSWs with other techniques may enhance effectiveness [18].

Effectiveness and acceptability of sitting-reduction interventions, more broadly, may also be enhanced by acknowledging how, why, and in what contexts office workers choose – or choose not – to stand. For example, although generally acceptable, desk-mounted SSWs limit space and pose practical problems for paper-based work (Chau et al., 2014). Studies have documented apprehension about colleagues’ potentially discouraging reactions from colleagues [11–13, 20]. People appear less likely to stand in meetings about sensitive topics, for example, to avoid detracting from the seriousness of the meeting [11].

Office workers’ responses to sitting-reduction strategies can reveal not only engagement with those strategies, but also broader barriers and facilitators of implementing sitting-reduction. The present study focuses on office workers’ experiences of attempts to limit sitting in response to an intervention prototype. The intervention aimed to reduce sitting and increase standing via feedback on sitting time, a range of tailored sitting-reduction techniques, and a desk-mounted SSW. Although originally designed to assess the acceptability of intervention components [21], our data transpired to predominantly offer insight into how office workers seek to reduce sitting and increase standing within the constraints of their working practices. While we also report intervention engagement, the main research question that guided the present analysis was: *how did office workers experience their attempts to reduce sitting?* The study was registered (ISRCTN29395780). Deviations from our published protocol [21] are detailed in Additional file 1.

### Method

### Participants, design and procedure

Office workers were recruited from a UK university (*n* = 29), between November 2016 and March 2017, to a 13-week uncontrolled intervention study. Sample size was determined by a predetermined recruitment window, constrained by funding. The study was advertised via posters at the host organisation, and fortnightly through all-staff circular emails throughout the 5-month recruitment period. The study was presented as an opportunity ‘to improve your workplace health, try out a sit-stand desk, and earn a £100 Amazon voucher’. Inclusion criteria required participants to: be aged 18 years or over; work at least 3 days per week; and spend most of their typical working day seated at a workstation, of which they were the sole user. Workers were not eligible where they: reported a physical condition prohibiting standing for prolonged periods; had previously participated in workplace standing research; ever used an SSW for two or more consecutive days; or intended to be absent for 10 consecutive workdays or leave the employ of the host organisation during the study period.

All those who expressed interest and were eligible attended the Preliminary Session, at which they provided consent and self-reported demographic characteristics (Table 1) and were fitted with an accelerometer-inclinometer device for 7-day continuous wear. Ten days later, they completed the Intervention Session at which they received accelerometry feedback, tailored advice, and a height-adjustable SSW. They were fitted with an accelerometer-inclinometer for further 7-day wear at the close of the Intervention Session, and 5- and 11-weeks post-intervention. Accelerometer data were collected and a semi-structured interview was conducted at one, 6 and 12-weeks post-intervention. Participants received a £100 ($125) Amazon voucher on study completion.

**Table 1:** Summary of participant characteristics

	*N (%)*
Gender	
Male	8 (28%)
Female	21 (72%)
Age	
18–24	3 (10%)
25–29	5 (17%)
30–34	6 (21%)
35–39	2 (7%)
40–44	3 (10%)
45–49	3 (10%)
50–54	4 (14%)
55–59	3 (10%)
60+	0
Ethnicity	
White	21 (72%)
Black / Black British / African / Caribbean	3 (10%)
Asian / Asian British	3 (10%)
Mixed ethnic background	2 (7%)
Monthly income	
£1500-2400	8 (28%)
£2400-3900	14 (48%)
> £3900	6 (21%)
Not reported	1 (3%)
Highest qualification	
A Level, AS Level, CSE, or GCSE	3 (10%)
Other technical or professional	2 (7%)
Degree or higher	22 (76%)
Non-UK qualifications	1 (3%)
Other	1 (3%)

All procedures, which were approved by the King’s College London Psychiatry, Nursing and Midwifery Ethics Panel (LRS-16/17–3718), were administered to each participant individually in a private room at their workplace by SD. SD is a male post-doctoral researcher with an experimental psychology background, and quantitative and qualitative data collection and analysis experience.

### Intervention

#### Preliminary session

Participants were fitted with an activPAL accelerometer-inclinometer (PAL Technologies, Glasgow, UK) using standard protocol [6]. activPALs are posture-sensitive, and reliably distinguish sitting, standing, and stepping [22]. Participants were asked to monitor their work tasks over the following 7 days, using self-generated task categories (e.g. ‘phone calls’, ‘word processing’). Tasks were recorded via replies to twice-daily emails from the researcher. On the final monitoring day, participants were also asked to estimate their total sitting time for each workday (9 am-5 pm) over the 7-day period.

#### Intervention session

Ten days after the Preliminary Session, the researcher met each participant in a private room at their workplace, to administer the intervention.

#### Sitting time feedback

Visual and verbal personalised feedback on sitting patterns during the monitoring period (i.e., ‘-1–0 weeks’ [baseline]) was provided and discussed in comparison to self-estimated sitting time.

#### Tailored behaviour change guidance

Next, participants were asked which one of three statements, representing the fundamental determinants of behaviour [23], was most diagnostic of them: “I do not feel capable of reducing my sitting at work” (capability); “I do not feel I have the opportunity to reduce my sitting at work” (opportunity); “I do not feel motivated to reduce my sitting at work” (motivation; all response options were ‘yes’ [most applicable] or ‘no’). Next, they chose from a menu of behaviour change strategies linked to their diagnostic statement. Five strategies (of which two were each offered only to those selecting one other strategy) targeted capability; three motivation; and one opportunity (Table 2). Following spoken delivery of chosen strategies, participants could choose advice relating to any other strategy, regardless of their diagnostic statement. Participants could choose as many strategies as desired.

**Table 2:** Behaviour change advice delivered in the Intervention Session

*Behavioural determinant targeted*	*Behaviour change strategy*	*Description of advice*	*Frequency with which chosen* *(Total N = 29)*
Capability	*Goal setting*	Guidance in setting specific and achievable behavioural goals for time spent sitting, standing and/or in light activity	14 (48%)
	*Action Planning*	Guidance in identifying specific contexts most conducive to sitting less, and developing ‘if-then’ plans for reducing sitting	10 (35%)
	*Habit Formation* *(only offered to those selecting Action Planning)*	Summary of psychological theory and evidence around how actions (e.g. sitting) become habitual via context-dependent repetition of the action	10 *(100% of those choosing Action Planning)*
	*Problem Solving*	Guidance on shielding an intended action (e.g. standing) from derailment in specific contexts, e.g., by identifying barriers and developing strategies to overcome them	1 (3%)
	*Habit Disruption* *(only offered to those selecting Problem Solving)*	Summary of psychological theory and evidence around how to obstruct unwanted habitual responses, either by avoiding cues (e.g. putting barriers in place) or adopting strategies to enhance likelihood of wanted response to habit cues (e.g., point-of-decision reminders)	1 *(100% of those choosing Problem Solving)*
Motivation	*Information on Health Consequences*	Detailed summary of evidence around health risks of sitting and benefits of standing and light activity	1 (3%)
	*Information on Others’ Experiences*	Testimonies from workers who had attempted to reduce sitting and increase standing in the workplace, derived from previous qualitative studies of sitting reduction, and descriptions of famous standing-workers (e.g. Dickens, Hemingway)	3 (10%)
	*Common Misconceptions*	List of potentially detrimental misconceptions about reducing sitting in the workplace, paired with evidence-based rebuttals	1 (3%)
Opportunity	*Tips for Standing*	Tips for incorporating more standing in to the workday: speaking to colleagues in person rather than emailing; standing in meetings; standing on the phone; walking during lunch; taking the stairs	4 (14%)
Various	*Tips for SSW use*	Tips for increasing likelihood of (ergonomically-sound) SSW use: leave the unit in standing position when leaving the office; move office chair away or cover with objects; increase SSW use gradually; ensure correct standing posture; shift weight from foot to foot; wear flat shoes or go barefoot while standing	*Compulsory (delivered to all participants)*

#### Sit-stand workstation

Lastly, a height-adjustable VariDesk Pro Plus 30 desk-mounted unit (Varidesk, TX, USA; £325 [US$405])) was fitted to the participant’s desk for the 12-week period. Participants were given ergonomic instructions and accompanying tips to promote frequent, ergonomically-sound SSW use (Table 1). Participants were permitted to retain the SSW indefinitely after participation, but this was only revealed to them upon study completion, so did not represent an active intervention component.

#### Reminders of intervention content

Participants were given the option of receiving email reminders over the 12-week intervention period of the key points from the Intervention Session. Participants specified the desired content and receipt frequency of these emails. All participants were emailed a summary of key points from the Intervention Session one day after that session.

### Data collection and analysis

#### Quantitative data: intervention engagement

Engagement was explored by describing the frequency with which each of the behaviour change strategies was selected, and inspecting sitting and activity levels over time. Accelerometry data on sitting, standing, and stepping time from the 5 workdays within the 7-day wear period, as measured between the Preliminary and Intervention Sessions (− 1–0 weeks [baseline]), and at 0–1, 5–6, and 11–12-week post-intervention, were extracted using specialist software (activPAL™ Professional v7.2.32; PAL Technologies Ltd., Glasgow, UK). A considerable amount of data at each timepoint were missing due to malfunctioning devices. A repeated-measures mixed-effects model assessed sitting, standing and stepping time changes, using study period (i.e. -1–0, 0–1, 5–6, 11–12-weeks) as predictor, and data for each of the 5 workdays in each period as covariates. Effect sizes for mean differences from baseline (Cohen’s d) were calculated for descriptive purposes.

#### Qualitative data: experiences of attempts to reduce sitting

Each participant was invited to take part in three one-to-one, face-to-face semi-structured interviews (at 1, 6, 12 weeks post-intervention), which explored: expectations and experiences of implementing the chosen strategies and of sitting and standing more broadly; SSW use; and the conduciveness of social and physical environments. Later interviews focused more on maintenance. Interview schedules are presented in Additional file 2. Interviews were audio-recorded and transcribed verbatim. Across the three timepoints, interview duration ranged from 9 to 43mins (mean 18mins). Twenty-one (72%) participants completed all three interviews, and eight (28%) completed only the 1- and 6-week post-intervention interviews, citing lack of availability for the third interview. Pertinent utterances within the Intervention Session, recorded in note form by the researcher, were also used as data.

All available qualitative data were analysed using realist inductive Thematic Analysis procedures [24]. Two coders (SD, BG) independently preliminarily analysed all data, involving data familiarisation and assigning labels to pertinent events. Comparison of notes between coders informed development of a thematic framework, which guided more in-depth coding conducted by BG. Themes were labelled using ‘in vivo’ codes to ensure they were grounded in real-world participant experiences. A third researcher (JH) inspected the framework and data excerpts and agreed that the analysis was supported by the data.

## Results

### Sample description

Of 29 participants, 21 (72%) were female. Age ranged from 18-24y to 55-59y, and monthly income ranged from £1.5–2.4 k to ≥£3.9 k. Most were White (21; 72%), and 22 (76%) had a university degree or higher. Seventeen people (59%) were in administrative roles. At baseline (− 1–0 weeks), mean sitting time was 355mins/workday (5 h 55 m; 74% of 8 h workday), standing time 82mins (17%), and stepping time 43mins (9%).

### Quantitative analyses: intervention engagement

#### Selection of techniques

Most participants (23/29; 80%) stated that none of the capability, motivation or opportunity statements applied to them, as they were sufficiently able and motivated, with enough opportunity to reduce sitting. Of the remainder, two stated that they most lacked capability (one lacked physical and social capability, the other psychological capacity), two most lacked motivation, and two most lacked perceived opportunity due to busy working schedules. Nonetheless, when invited to select from all strategies, 26 participants (90%) chose to receive advice on at least one strategy. Twenty participants (69% of sample) chose Goal Setting or Action Planning; six chose both, eight chose Goal Setting only, and six Action Planning only (Table 2). All who chose Action Planning also opted for Habit Formation advice.

#### Behavioural responses

Study period predicted sitting time (F[3, 122.5] = 28.9, *p* < .001): relative to baseline (− 1–0 weeks), sitting time reduced by 50 min at 0–1 week, 118 min at 5–6 weeks, and 110mins at 11–12 weeks, by which point mean sitting time (245 m; 4 h 5 min) represented 51% of the 8 h workday (Table 3). An equivalent increase was observed in standing time (+ 49 m at 0–1 week; + 116 m, 5–6 weeks; + 113 m, 11–12 weeks; F[3, 120.7] = 31.1, p < .001), but there was no change in stepping time (F[3, 138.1] = 2.1, *p* = .10).

**Table 3:** Sitting, standing and stepping time (in mins) per 8 h workday, across study timepoints

	Baseline (−1–0 weeks) *N = 29*	Post-intervention 1 (0–1 weeks) *N = 21*			Post-intervention 2 (5–6 weeks) *N = 18*			Post-intervention 3 (11–12 weeks) *N = 16*		
	*Mean (SD)*	*Mean (SD)*	*Mean difference from baseline* ^a^ *(SD)*	*Cohen’s d*	*Mean (SD)*	*Mean difference from baseline* ^a^ *(SD)*	*Cohen’s d*	*Mean (SD)*	*Mean difference from baseline* ^a^ *(SD)*	*Cohen’s d*
Sitting time	355 (14)	305 (18)	−47 (82)	−0.50	237 (17)	−101 (103)	−1.13	245 (20)	−100 (100)	− 1.08
Standing time	82 (13)	131 (17)	51 (75)	0.56	198 (16)	101 (94)	1.17	194 (20)	101 (99)	1.11
Stepping time	43 (3)	4 (21)	−5 (21)	−0.22	45 (4)	0 (18)	−0.01	40 (4)	0 (14)	0.03

^a^Mean differences from baseline pertain to participants for whom data were available at each timepoint. For this reason, values do not correspond to the difference between the mean of each baseline timepoint (Ns < 29) and the baseline mean where N = 29

### Qualitative analyses: experiences of attempts to reduce sitting

Five themes related to: motives, expectations and outcomes; physical and practical challenges; social dynamics; counter-motives and use of cues; and routinisation.

**“I sit at my desk an awful lot”: Motives, expectations and outcomes**

Most people entered the study to trial the sit-stand workstation (SSW). Many appeared aware of possible health benefits of displacing sitting with standing, such as alleviation of existing health problems, or avoiding deterioration of health. Some saw the intervention as a cue to acting on prior motivation:

Participant 11, Interview 1 (P11, I1): *I do sit at my desk an awful lot and I … know that that’s not good for my health, so anything that … gives me a nudge to actually do something about it is bound to be good.*

Most were strongly motivated to stand, and felt physically capable of standing, though some felt trepidation about responses from co-workers for contravening workplace norms (“*it’s that sense [of] is that acceptable, for this person [to be] doing it differently?”*; P5, I1).

Others worried that working at a different height to other colleagues “*might be quite irritating”* (P6, I1) or intrusive (“*do they think you’re looking over their shoulder or something?”;* P1, I1), or suggestive of an undesired social identity (“*there’s the perception that standing desk people are … trendy, health-conscious people”;* P5, I1)*.*

The intervention was perceived as beneficial in multiple ways. Accelerometry feedback raised awareness of true sitting patterns (“*it was quite shocking … when I realised how much time [I had sat]”*; P14, I1)*.* Many participants reported increased standing time, mostly due to the SSW. Some reported that standing spurred further movement (“*[I’m] more likely to move to another bit of the office … because I’m already standing”;* P27, I1). For some, the intervention instilled a ‘sit less, move more’ mindset, characterised by greater awareness and use of opportunities for reducing sitting at work and elsewhere:

P5, I3: *[On the train] even if there’s a seat available, I think, ‘Oh, I’ll just stand’, not only because the journey is not going to be that long, but also I should just stand, it’ll be healthier, I don’t need to sit down. […] That thought has occurred to me more since starting [the intervention].*

Participants attributed improvements in posture, strength and balance (“*since I’ve started with the desk I can stand with minimal or no wobble*”; P16, I2), and reductions in pain (“*I’m not getting as much backache as I used to*”; P3, I2), to increased standing.

Some felt that standing boosted alertness, in turn increasing productivity (“*I feel a lot better and I do feel energised*”; P4, I1), though some felt it had no impact (“*I’m not fussed whether I stand up or sit down […] but I like to have the option*”; P22, I3).

**“It’s been a lot more tiring than I thought”: Physical and practical challenges**

While some reported less fatigue than expected (“*I thought my back would be sore … but actually it’s been absolutely fine*”; P22, I3), several participants experienced unanticipated physical fatigue from standing (*“[it’s] been a lot more tiring than I thought*”; P28, I1). While discomfort often prompted participants to sit, most reported fatigue diminishing as they gained experience (“*I grew accustomed to how it would feel*”; P8, I3).

Participants reported various practical barriers to SSW use, which many felt could not easily be used with equipment essential for work tasks, or for paper-based tasks. Some were able to adapt to the constraints imposed by the workstation:

P17, I2: *I might put paperwork on the bottom bit of the desk and my keyboard on the higher bit if I’m not using it as much, [or] sometimes I … put paperwork on my chair. […] I haven't found that there’s anything where I can’t stand.*

**“Everybody’s been really interested”: Social dynamics.**

Despite prior concerns, most experienced encouragement from colleagues. Some felt minimally self-conscious because their workstation lacked visibility (“*I’m out of the way … if I were standing up in the middle [of the open-plan office], I’d feel an idiot*”; P6, I2), or because they had explicitly gained approval to stand (“*I said ‘I’m not going to sit with you if that’s alright, I’m going to carry on standing’ [and] they went, ‘that’s fine*’”; P4, I1). Several people reported that standing, and particularly the SSW, facilitated interaction:

P15, I1: *Everybody’s been really interested … they’re saying ‘oh, that’s cool’. The novelty helps in terms of the motivation.*

Some felt more psychologically comfortable being approached by others when standing, which created more equitable power relationships (“*I enjoyed the aspect of being on the same equivalent level and eye level”*; P19, I3)*.* Some found standing ‘*empowering’* (P13, I1) when making phone calls:

P11, I1: *There’s this tiny little bit more confidence [when] standing up with [voice] calls … I feel as though I’m towering over them.*

Several people found standing conducive to collaboration when colleagues gathered around the SSW, due to greater monitor accessibility (“*it’s really good if you are both standing instead of huddled over at a computer”*; P14, I3). Enhanced visibility when standing could however compromise privacy. Several participants reported that colleagues were more likely to interrupt them (“*you’re more approachable [when standing]”*; P19, I3), and some were more distracted by others’ activities, when standing:

P14, I2: *When I’m standing up … if there’s something going on … I hear a bit better, and hear something else going on, whereas when I’m sitting I’m more likely just to hear it but then carry on with my work.*

**“Now is a good time”: Counter-motives and cues to standing.**

Participants cited multiple factors that could derail standing. Many found it psychologically effortful to raise the SSW, which precluded short standing bouts (P7, I3: “*If I’ve only got a brief period of time […] it seems an awful lot of effort to stand up”*). Tiredness also limited motivation (“*I wasn’t sleeping properly […] [it felt like] an effort to be at work, let alone also stand up*”; P16, I3), though some stood to offset postprandial tiredness (“*I’ll hoik [the workstation] up and it’ll give me a bit more energy*”; P9, I2).

Participants’ primary motivation was to complete work tasks, so they did not stand where it was seen to conflict with working (P7, I3: *“I need to do what I need to do, work has to come first”*). Being engrossed in work led to forgetting to stand. For one person, sitting was comforting during stressful periods:

P21, I3: *[My job] is high pressure the whole time, and so … I feel a bit sorry for myself and sitting down is like a treat.*

Time cues were effective for some (P1, I1: “*I just put a timer on my phone and I reset it every half-hour and I go up and down*”), but many people ignored them because they suggested action at moments when standing was not prioritised:

P23, I2: *I had my reminders on my watch which continually told me to stand, but I found myself turning that off … because I got caught up with other things.*

For most, completion of a work task acted as a convenient and salient cue to stand (a “*natural break point*”; P5, I1). Many described a “*sorting out*” period (P26, I2) upon arriving at work, characterised by answering emails and mentally preparing for the day ahead, completion of which commonly cued standing:

P25, I3: *I’ve checked my emails, done all that sort of thing. … Once I have got my brain into the tasks for today then I’ll do the standing [and] get down to the nitty-gritty of the work.*

Some chose to stand after lunch to aid digestion:

P19, I1: *You have your lunch, feel a bit lethargic, and then it’s nice to stand. It’s almost working that lunch off. I enjoy that.*

Participants were also cued to stand when expecting to perform certain tasks. Most felt standing was ill-suited to cognitively-intensive tasks (“*if I need to really concentrate on something then sitting is better*”; P9, I1), so chose to stand for routine tasks (“*this morning I was just sending emails and looking at stuff, it was easy to stand*”; P6, I1). Some deemed standing helpful for maintaining focus when performing less cognitively engaging tasks. Others reported that switching from sitting to standing provided valuable ‘thinking space’:

P14, I3: *I was having some issues so then I stood up and it just woke me up a bit I suppose. So instead of the monotony of just sitting there trying to work a problem out, it was quite good to stand up and almost look at it differently from [a] standing [perspective].*

Where participants could not rely on external cues due to variable work patterns, standing was inconsistent and sporadic.

**“Getting into a rhythm”: Routinisation of standing**

Most participants incorporated some standing into their working routine. Routinised standing was characterised by lesser mental effort (“*it’s part of my routine now … it’s not a chore for me*”; P24, I1), and reduced reliance on external cues (“*I just know that when I’m coming in [to the office, the SSW] is going up*”; P9, I1). Several people adapted to the workstation over time, becoming able to complete most tasks standing (“*I have started to do more tasks standing up, whereas before it was [only] repetitive things*”; P14, I2), and could become *“completely absorbed [in work] and forget that I’m standing”* (P25, I2). Routinised standers used physical discomfort as a cue to stand.

Newfound standing routines were liable to disruption, due to absences from work, or changes in workload. Some struggled to re-establish standing after such disruption and found that standing became physically arduous again. While some participants effortfully but successfully recovered standing after such disruption, others lapsed into old sitting habits:

P23, I2: *A week’s holiday and then a period of just meetings after meetings pretty much every day, and at that point [my standing] kind of declined. I got to a stage … where I thought oh God, I actually haven’t stood … properly for a week. It felt like I had gone right back to the beginning again.*

## Discussion

This study of 29 office workers explored experiences of a workplace intervention comprising a sit-stand workstation (SSW) and tailored advice. Sitting time reduced from baseline by 50mins at 1-week, 118mins at 6-weeks, and 110mins at 12-weeks post-intervention. This corresponded with increases in standing and at 12-weeks mean standing time was 3 h 14 min, firmly achieving the 2-4 h recommendation [7]. Interviews provided important insight into contextual factors that shaped participants’ experiences. Findings support further development of our intervention and generate broader design and implementation recommendations for workplace sitting-reduction interventions.

Although only a small uncontrolled study, observed reductions in sitting time justify further development and testing of our intervention. Qualitative data pointed to potential reasons for declines in sitting. Device-based feedback raised awareness of sitting, in turn leading people to adopt a ‘sit less, move more’ mindset both in the workplace and elsewhere (e.g., when commuting; [25]). This testifies to the lack of attention people pay to sitting and suggests an ‘audit and feedback’ approach may motivate sitting reduction.

Few participants reported deficiencies in motivation, capability, or opportunity at the study outset. Nonetheless, most opted to receive advice on goals and planning, implying that they expected to reduce their sitting most if they were more psychologically capable, or better able to capitalise on opportunities. Qualitative data highlighted the potential for unforeseen barriers to impact on attempts to sit less, apparently by diminishing capability or perceived opportunities. One such barrier was physical effort; several participants found standing more taxing than anticipated [11], though physical fatigue diminished over time for some. Sitting-reduction interventions might manage expectations by highlighting the possibility of mild discomfort and offering mitigating strategies. Participants could use discomfort as a cue to transitioning not only from sitting to standing, but also from standing to sitting.

Our data support previous studies in suggesting that people find some tasks less suited to standing [13, 25, 26]. While there were predictable practical barriers to SSW use (e.g. making calls from a wired phone [25]), we also observed important psychological barriers. The perceived mental effort involved in raising the SSW was, for some, only deemed worthwhile for lengthy tasks, and some participants preferred to stand only for less cognitively involved tasks. This supports the perspective that the postural allocation system that regulates standing draws on the same finite resources as mental processes, such that standing impairs performance of cognitively demanding tasks [27]. People can perform simple motor tasks (e.g., typing) as effectively when standing or sitting [28], but the impact on more mindful tasks (e.g., writing reports) has not been evaluated. Interestingly, some participants reported becoming able to perform more demanding tasks while standing. This suggests either that, as people grow accustomed to standing, they can incorporate tasks that are inherently more difficult to perform while standing, or that there is no inherent disadvantage to completing such tasks while standing. Some people valued breaking up sitting as a means of achieving mental ‘space’ to solve problems. The perceived suitability of tasks to standing may therefore be based on personal preference. Interventions should encourage workers to identify tasks they feel most able to complete while standing.

Some moments may be more opportune for standing. Participants prioritised work tasks over sitting or standing and preferred to change posture upon completing discrete tasks. Theory offers two possible reasons for this preference: people may be more likely to attend to their surroundings at the boundary between one task and another, making the need to stand more salient, or they may be less willing to stand mid-task because they find it distracting [29, 30]. Interventions should acknowledge how people segment their day or workload into discrete ‘units’, as these may represent ‘natural break points’ for standing. We identified several such points, such as the completion of a period involving ‘clearing’ work accrued since the previous workday or returning from time away from the desk. Interventions will be less intrusive, and perhaps more effective, if they promote sit-to-stand transitions at points at which workers are most psychologically capable of standing. Identifying reliable contexts for consistent standing may also foster habit, whereby standing at opportune moments becomes an automatic response that requires little forethought or conscious effort [31].

Although participants voiced trepidation about others’ responses [11, 12, 32], these concerns were typically not realised. Standing conferred some unexpected social benefits: some found it empowering, and the SSW facilitated social interactions and collaborations [25], though some deployed the SSW to create personal space and minimise distractions. Although further work is however needed to more comprehensively document the social functions of SSWs, concerns about others’ responses may be minimised via obtaining organisational support for sitting-reduction interventions, to demonstrate explicit social approval for attempts to sit less and stand more [14].

Limitations must be acknowledged. Our participants’ experiences were specific to our intervention prototype and may have varied had we adopted different intervention content or delivery methods. In particular, the SSW used – a desk-mounted unit that allows for the computer keyboard and monitor to be raised, rather than an adjustable sit-stand desk that raises the entire desk-top – limited the appeal of standing for tasks that required desk space [13]. We focused only on the experiences of intervention recipients, but successful implementation also requires addressing concerns among management, which typically focus on the effects of standing on productivity [12, 14, 33]. There is growing recognition of the importance of targeting sitting-reduction at both individual and organisational levels [14, 18].

Sample characteristics may also reduce generalisability. Many participants entered the study to trial an SSW, suggesting prior sitting-reduction motivation, and most were female, and highly educated, which limits the representativeness of the experiences of our sample. While anecdotal feedback from participants suggested that few were consciously motivated by the incentive of a £100 voucher conditional on study completion, this may nonetheless have sustained engagement with the intervention. Additionally, the same researcher delivered the intervention and conducted interviews, so participants may have been unwilling to disclose negative views or non-adherence. The intervention was delivered face-to-face, a time- and resource-intensive format unlikely to be scalable ([12]). Our subsequent work will refine intervention content for delivery in an alternative format.

## Conclusions

This study showed our intervention prototype to be promising, and moreover yielded insight into experiences of implementing sitting-reduction advice into workplace routines. Next, we will refine our intervention for self-administration as an online staff-training module, a common workplace education and training delivery format. Future interventions should acknowledge the barriers and facilitators of sitting-reduction we have documented.

## Additional files

Additional file 1: Deviations from protocol. (DOCX 15 kb)

Additional file 2: Interview schedules. (DOCX 22 kb)

## Data Availability

Study data are available from the corresponding author on request.

## Abbreviations

I: Interview number
P: Participant
SSW: Sit-stand workstation
