# Supplementary material for: Office workers’ experiences of attempts to reduce sitting-time: an exploratory, mixed-methods uncontrolled intervention pilot study
Source: BMC Public Health. 2019 Jun 25;19:819. doi: 10.1186/s12889-019-7196-0 (PMC6593587; doi:10.1186/s12889-019-7196-0)
Supplement: Supplementary file 1 — Deviations from protocol. (DOCX 15 kb) [file 12889_2019_7196_MOESM1_ESM.docx]

*Deviations from protocol*

Data collection and analysis deviated from our published plan as follows [21]. We mistakenly stated in the protocol that the Intervention Session was audio-recorded; Intervention Session data were recorded only via researcher notes.

Many participants reported removing the accelerometer for the weekend period, or at the end of the working day, and reattaching it only for workday wear. We had intended to analyse accelerometry data over 7 waking days (0700-2300), only for participants with at least 3 weekdays and 1 weekend day of 24h wear, but this would have removed most available data. Hence, analyses are based only on available data during the assumed working day (0900-1700 [8h = 480mins] over 5 days).

Qualitative analyses were designed to reveal the acceptability of intervention components. However, interview data offered minimal insights into discrete components other than the SSW, possibly because participants did not recall or feel strongly towards chosen strategies. The dataset was, however, richly illustrative of attempts to reduce sitting and increase standing, so our analysis focuses on barriers and facilitators of modifying sitting and standing at work, which may inform intervention development beyond our own project.
